# Raffinose family oligosaccharides (RFOs): role in seed vigor and longevity

**DOI:** 10.1042/BSR20220198

**Published:** 2022-10-04

**Authors:** Prafull Salvi, Vishal Varshney, Manoj Majee

**Affiliations:** 1 National Agri-Food Biotechnology Institute, Punjab 140308, India; 2 Govt. Shaheed Gend Singh College, Charama, Chhattisgarh 494337, India; 3 National Institute of Plant Genome Research (NIPGR), Aruna Asaf Ali Marg, New Delhi 110067, India

**Keywords:** Galactinol, plant biology, RFO, seed

## Abstract

Seed vigor and longevity are important agronomic attributes, as they are essentially associated with crop yield and thus the global economy. Seed longevity is a measure of seed viability and the most essential property in gene bank management since it affects regeneration of seed recycling. Reduced seed life or storability is a serious issue in seed storage since germplasm conservation and agricultural enhancement initiatives rely on it. The irreversible and ongoing process of seed deterioration comprises a complex gene regulatory network and altered metabolism that results in membrane damage, DNA integrity loss, mitochondrial dysregulation, protein damage, and disrupted antioxidative machinery. Carbohydrates and/or sugars, primarily raffinose family oligosaccharides (RFOs), have emerged as feasible components for boosting or increasing seed vigor and longevity in recent years. RFOs are known to perform diverse functions in plants, including abiotic and biotic stress tolerance, besides being involved in regulating seed germination, desiccation tolerance, vigor, and longevity. We emphasized and analyzed the potential impact of RFOs on seed vigor and longevity in this review. Here, we comprehensively reviewed the molecular mechanisms involved in seed longevity, RFO metabolism, and how RFO content is critical and linked with seed vigor and longevity. Further molecular basis, biotechnological approaches, and CRISPR/Cas applications have been discussed briefly for the improvement of seed attributes and ultimately crop production. Likewise, we suggest advancements, challenges, and future possibilities in this area.

## Introduction

Almost every living organism is directly or indirectly dependent on the plants or plant products for their survival. For a human, the agriculture sector is the principal means of livelihood, and thus the vigor and vitality of plants particularly seeds is certainly one of the most crucial factors. Plants have a varied life cycle with a wide range from centuries to a few weeks [1]. Interestingly, seed occupies a crucial position in the life cycle of plants and possesses a number of spectacular characteristic features including their ability to survive for a prolonged period in a desiccated state [2]. The longevity of orthodox seeds can range from decades to centuries and even millennia (*Phoenix dactylifera*: >2000 years and *Nelumbo nucifera*: over 1300 years) [3,4]. Seed dormancy is another important feature associated with germination, vigor, and viability [5]. In general, dormancy refers to the metabolic arrest state (or reduced metabolic rate) of an organism in which the growth, development, and physical activities cease temporarily to preserve energy and promote survivability under harsh environmental conditions. Seed dormancy is crucial from an agriculture point of view; thus, it is a most studied aspect of seed physiology [2,6]. However, it is imperative for plants to have an adequate balance of seed dormancy as higher dormancy is undesirable when rapid and synchronized germination is required [2,7–9]. Likewise, the reduced dormancy will have a preharvest sprouting phenotype, which also results in an economically undesirable trait [2]. The timespan from dormancy to germination period varies among species, and thus the seeds need to remain alive for a long time, i.e., higher longevity which relies on the seed vigor. In simple words, seed longevity can be understood as the entire life span of a seed (after dry storage) till it remains viable. Seeds are also resistant to environmental stresses, which allow them to survive in extreme weather. Seed longevity is a complex trait and the vigor and viability of seeds are influenced by the moisture content, temperature, or the environmental cues they encounter [9–14]. As per research, the seed has to perform complicated processes such as protection, repair, and detoxification, to maximize its life span or viability [15–17]. However, the underlying molecular complexities and processes of seed germination vigor and viability over extended durations remain unknown. Though, in the last few years, various mechanisms, and key proteins have been identified that play a significant role in preserving seed vigor and longevity. Among them, a protein repairing enzymes protein l-isoaspartyl methyltransferase (PIMT), an evolutionary conserved ancient enzyme emerged as a key factor in seeds, which plays a predominant role in preserving seed vigor and viability for prolonged periods of time [18–20].

Parallelly, carbohydrates and/or sugars have emerged as promising components and have shown to be involved in regulating seed vigor and longevity in recent decades. Carbohydrates are the fundamental cellular elements, characterized by the basic chemical formula [C_*x*_(H_2_O)_*y*_], and contain carbon hydrates [21,22]. Mainly, sugars are polyhydroxy aldehydes or ketones, that have been classified largely by molecular size, individual monomer properties, degree of polymerization (DP), and type of linkages [23]. Plants usually store carbon as starch and translocate it as sucrose [24]. However, several plant species can retain and distribute alternative carbohydrates such as raffinose family oligosaccharides (RFOs) [6,22,25,26]. RFOs are transported through phloem in the members of the family Cucurbitaceae, Lamiaceae, Oleaceae, and Scrophulariaceae, which form a symplasm with mesophyll and sieve components [27,28]. RFOs are stored carbohydrates in seeds, and one of their hypothesized purposes is to provide immediately available energy for seed germination [6]. However, the role of RFOs has been widely investigated in several plant physiology and cellular functions like signal transduction, carbon storage, photoassimilate translocation, membrane trafficking, biotic and abiotic stress tolerance, mRNA export, and transport, seed desiccation tolerance, and seed germination [29–35]. Likewise, the role of RFOs in controlling plant seed traits related to seed vigor and longevity [6,35,36] is also reported, and this area has the potential to explore further for better understanding.

Hence, this review emphasizes and provides updated, succinct knowledge about the significance of RFOs in seed physiology, namely seed germination, vigor, longevity, and desiccation tolerance. This review paper also highlights some of the key regulatory aspects of RFO pathways, as well as the problems and barriers to extending seed longevity. We also talked about the several biotechnological approaches and CRISPR/Cas applications to boost seed vigor and longevity, which leads to crop improvement and ultimately sustainable agriculture. In addition, potential scientific challenges and future research directions in seed biology have been discussed.

## Metabolic and molecular fluctuations during seed aging

Seed longevity is a complex trait, which is influenced by several factors, among them genetic variations hold a remarkable impact and thus attribute to the differential longevity among organisms or genotypes [10]. Previously, the oldest viable seed was considered to be date palm seed (*Phoenix dactylifera*), which showed germination even after 2000 years (yrs) [4]. However, several other species also showed extreme longevity with the ability to germinate after 500–10,000 yr, e.g., *Lupinus arcticus* (10,000 yrs), *Nelumbo nucifera* (1300 yrs), Achira seed (550 yrs), etc [3,37]. Interestingly, a decade ago, Yashina et al. [2012] reported the *Silene stenophylla* is the oldest seed buried in Siberian permafrost, which retained germination potential after 30,000 yrs and regenerated into an entire fertile plant [38]. It is worth noting that such extreme longevity displayed by these seeds is not solely attributed to their genotype, rather, their environmental aspects like moisture content, oxygen level, temperature, light intensity, etc. are equally important. Being a complex trait, seed longevity is governed by many genes and thus strongly influenced by environmental factors encounter during seed development, seed-set, harvest-time, and their storability [39].

**Figure 1:**
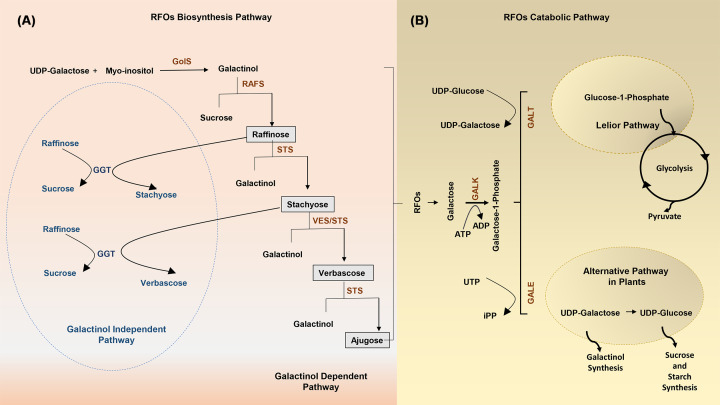
Schematic pathway of RFO family metabolism in plants (**A**) RFOs biosynthesis pathway: RFO biosynthesis is initiated by the conversion of Myo-inositol and UDP-Galactose into galactinol utilizing GolS. The galactinol further acts as galactosyl moiety donor for generation of successive RFO members like raffinose, stachyose, verbascose, and ajugose utilizing indicated enzymes in the pathway. This pathway is called galactinol-dependent. in galactinol independent pathway, GGT catalyses the direct transfer of the galactosyl moiety from one RFO molecule to another and leads to the formation of RFO members; GGT, galactan-galactosyltransferase; GolS, galactinol synthase; RAFS, raffinose synthase; STS, stachyose synthase; VES, verbascose synthase. (**B**) RFOs catabolic pathway: RFOs are digested or catabolized first into galatose-1-phsophate via GALK that further has two fates. One is Lelior pathway where it produces glucose-1-phosphate via GALT and other one is to undergo alternative pathway where the end products are UDP-glucose and UDP-galactose catalysed by GALE; GALE, UDP-galactose 4′-epimerase; GALK, galactokinase; GALT, galactose-1-phosphate uridyltransferase.

Desiccation tolerance (DT) is one of the key factors which greatly affect seed vigor and longevity. DT is established during the seed development or seed formation which is acquiesced through a cascade of different phytohormones, transcription factors, and the accumulation of protective molecules like RFOs [2,40–46]. During the dormant stage, a cellular system of seed is minimally active, which necessitates a high protective mechanism under such a dry state. The metabolic process remains temporarily switched off in order to preserve energy, and the seed majorly accumulates protective biomolecules like soluble sugars, antioxidants as well as protein/nucleic acid repairing enzymes [39,47,48]. According to free radical theory, the negative impact of free radicals on macromolecular functions including DNA/RNA damage, interfering protein synthesis, lipid peroxidation, etc are the key molecular mechanism underlying the aging process in all organisms [49,50]. Many detoxification proteins have been found that control *Oryza sativa* grain longevity, including phosphatidylinositol 3-kinase (OsPI3K), acetyl-CoA carboxylase (OsACCase), and aldehyde dehydrogenase7 (OsALDH7) [51–53]. Similarly, the roles of detoxifying proteins such as thioredoxin peroxidase (TPX), glutathione transferases (GST), glyoxylases (GLO), superoxide dismutase 4 (SOD4), and catalase 3 (CAT3) in *Zea mays* seed longevity have been found [1,54–56]. Despite a certain level of reactive oxygen species (ROS) being beneficial or essential for adequate functioning of cellular processes, the rise of ROS level above the threshold is concomitantly linked with cellular injury. Besides, mitochondria are a key site for ROS generation (mtROS), thus its altered intercellular redox status activates the gene regulatory response, which modulates the expression profile of nuclear and mitochondrial genes [50,57,58]. The increased ROS affects cellular functionality by oxidative damage to the crucial enzymes involved in ATP synthase, TCA cycle, and pyruvate decarboxylase to name a few [58]. These cellular enzymes are damaged at the amino acid via oxidation of the methionine (generate methionine sulfoxide) or cysteine (form disulfide bonds) or formation of the carbonyl group at the side chain of threonine, serine, lysine, arginine, etc [58–60] (Figure 1).

Accumulative evidence from genetic analysis of seed vigor and longevity in different crops has indicated the important role of RFOs in seed longevity [6,22]. Since during seed storage, the temperature and relative humidity (RH) enhance the aging process, thus aging can be induced artificially by challenging the seed to high temperature and RH under controlled conditions. Such exposure of seed to high temperature and RH is referred to as controlled deterioration treatment (CDT), which is reported in several studies to mimic the aging process [17,20,61,62]. Seeds cultivated under a greater ratio of far-red/red light circumstances were more tolerant to CDT than *Lactuca sativa* seeds grown under red-light conditions [63,64]. During seed aging, the macromolecules are impacted which results in compromised vigor. As previously noted, protein repair enzymes such as PIMT and methionine sulfoxide reductase (MSR) were shown to be involved in repair activities and played an essential role in boosting seed vigor and lifespan in many plant species [19,20,45,65–67]. PIMT, for example, decreases isoaspartic acid (IsoAsp) and modulates ROS homeostasis in seeds [20,45,68], whereas MSR reduces sulphoxide ions, which ultimately enhances the seed life by reducing the ROS toxicity [67,69].

Notably, the involvement of transcription factors such as abscisic acid insensitive 3 (ABI3), abscisic acid insensitive 5 (ABI5), leafy cotyledon 1 (LEC1), LEC2, and Fusca 3 (FUS3) in controlling seed viability and lifespan has been extensively researched, which indicates that seed maturation, vigor, and longevity is a complex and progressing process that is tightly regulated at multiple levels and by involving several mechanisms and pathways [47,70–73]. Likewise, late embryogenesis abundant (LEA) proteins and heat shock proteins (HSPs) play important roles in seed lifespan [47,61,74]. Overexpression of *HaHSFA9* from *Helianthus annuus* in *Nicotiana tabacum* seeds, for example, significantly increased seed vigor and longevity [74,75]. Further in the future, studies on several HSPs, as well as other potential genes, are being exploited as candidates for genetic enhancement of grain seed life.

Additionally, the physio-chemical properties of the seed coat also affect the seed longevity as the structural facets of the seed coat act immediate protective barrier for the embryo from microbial and other stressors [76,77]. While the seed coat prevents seed deterioration, it is also negatively correlated with germinability, as it restricts water uptake. For instance, the seed coat of transgenic soybean (*GmHs-1*) possesses a high concentration of calcium which result in a hard-seededness phenotype. The hard‐seededness of *GmHs-1* transgenics was associated with reduced germination and better longevity [76]. Similarly, the accumulation and distribution of secondary metabolites including polyphenols, isoflavones, anthocyanins, etc., to different parts of seeds positively correlated with seed longevity. Interestingly, a multi‐antimicrobial extrusion protein (MATE) transporter mutant in Arabidopsis (*at-tt12*) has compromised proanthocyanins accumulation and decreased seed longevity [78]. A high lignin (a phenolic compound) accumulation in the seed coat reduces the mechanical injury of the seed during transportation thus enhancing seed longevity [78]. Similarly, defense-related proteins are associated with seed life because they preserve seeds in dry conditions.

In general, the factors affecting seed aging are usually driven by the upsurge in ROS levels. Therefore, further deeper knowledge of the mechanism underlying would be required to understand the molecular and genetic components that are involved in seed vigor and longevity. Advanced molecular investigations and many mechanisms involved have revealed the underlying processes controlling seed vigor and longevity have been summarized in Table 1. The functions and mechanisms underpinning RFOs and sugars in enhancing seed vigor and longevity are also discussed in this review article.

**Table 1:** Genes and mechanisms involved in governing the seed vigor and longevity in plants

S.No.	Genes name	Mechanism involved	Organisms	References
1.	*AtOGG1* (DNA glycosylase/AP lyase)	DNA modification repair mechanism	*Arabidopsis thaliana*	[157]
2.	*AtPARP3* (Poly-ADP-ribose polymerase)	DNA modification repair mechanism	*Arabidopsis thaliana*	[158]
3.	*AtLIG4* and *AtLIG6* (DNA ligase IV)	DNA modification repair mechanism	*Arabidopsis thaliana*	[159,160]
4.	*MSR* (*Methionine Sulfoxide Reductase*)	Protein repair mechanism	*Medicago truncatula, Arabidopsis thaliana*	[67]
5.	*PIMT1* and *PIMT2* (*Protein-L -Isoaspartyl O -methyltransferase 1 and 2*)	Protein repair mechanism	*Arabidopsis thaliana, Oryza sativa, Cicer arietinum*	[18,19,45,66,161]
6.	*ROF1* and *ROF2* (*ROTAMASE FKBP 1* and *2*)	Protein repair mechanism	*Arabidopsis thaliana*	[162]
7.	*ASK13* (*Arabidopsis SKP1-like Protein 13*)	Ubiquitin–proteasome pathway	*Arabidopsis thaliana*	[163]
8.	*GolS* (*Galactinol Synthase*)	RFO metabolism mechanism	*Arabidopsis thaliana, Cicer arietinum*	[12,35,127]
9.	*Raffinose Synthase*	RFO metabolism mechanism	*Arabidopsis thaliana*	[36,101,164]
10.	*Stachyose Synthase*	RFO metabolism mechanism	*Arabidopsis thaliana*	[92,101,165]
11.	*α-Galactosidase*	RFO metabolism mechanism	*Arabidopsis thaliana*, Beech	[101,113,123]
9.	*AKR1* (*Aldo-Ketoreductase1*)	Lipid peroxidation mechanism	*Oryza sativa*	[166]
10.	*OsLOX2 (Lipoxygenase 2)*	Lipid peroxidation mechanism	*Oryza sativa*	[167]
11.	*OsPI3K* (*Phosphatidylinositol 3-Kinase*)	Detoxification mechanism	*Oryza sativa*	[53]
12.	*OsACCase* (*Acetyl-CoA carboxylase*)	Detoxification mechanism	*Oryza sativa*	[52]
13.	*OsALDH7* (*Aldehyde Dehydrogenase7*)	Detoxification mechanism	*Oryza sativa*	[51]
14.	*SOD4* (*Superoxide dismutase 4*)	Antioxidant mechanism	*Zea mays*	[168,169]
15.	*CAT3* (*Catalase 3*)	Antioxidant mechanism	*Zea mays*	[168,169]
16.	*TPX* (*Thioredoxin peroxidase*)	Antioxidant mechanism	*Zea mays*	[168,169]
17.	*GST* (*Glutathione transferases*)	Antioxidant mechanism	*Zea mays*	[168,169]
18.	*GLO* (Glyoxalase)	Antioxidant mechanism	*Zea mays*	[168,169]
19.	*HaHSFA9 (Heat Shock Factor9)*	Heat Shock Factor	*Helianthus annuus*	[170,171]
20.	*ABI3* (*Abscisic Acid Insensitive 3*)	Transcription factor	*Arabidopsis thaliana*	[172–174]
21.	*LEC1* and *LEC2* (*Leafy Cotyledon 1 and 2*)	Transcription factor	*Arabidopsis thaliana*	[175]
22.	*FUS3* (*FUSCA3*)	Transcription factor	*Arabidopsis thaliana*	[70]

## Raffinose family oligosaccharides metabolism

Next to sucrose, RFOs are soluble carbohydrates that are primarily found in higher plants [79]. RFOs are commonly found in the seeds of many crop plants, mainly belonging to the Leguminosae family, such as *Cicer arietinum* (chickpea), *Lens culinaris* (lentil), and *Glycine max* (soybean) [12,22,25,34,80]. They can also be accumulated in roots and specialized storage organs like leaves and tubers. RFO concentrations in *Stachys sieboldii* (Chinese artichoke) tubers and photosynthesizing *Ajuga reptans* leaves, for example, vary from 25 to 80% of their dry weight [6,79,81,82].

RFO biosynthesis is catalyzed by α-galactosyl transferases, which drive the sequential transfer of galactosyl moieties to the sucrose. In RFO biosynthesis, galactinol synthase (GolS) is the most crucial enzyme and the step it catalyses as it produces galactinol, which acts as a galactosyl donor for the generation of other members of RFOs [12,83,84]. Raffinose, the first member of RFOs is a trisaccharide synthesized from the action of raffinose synthase (RAFS), which utilizes sucrose and galactinol as substrates. Similarly, stachyose is produced after the transfer of galactosyl moiety to raffinose by stachyose synthase (STS) [85,86]. Further transfer of the galactosyl moiety on to this chain will produce higher RFOs members such as verbascose and ajugose [35,84,87]. The transfer of galactinol to stachyose results in verbascose synthase (VES) mediated verbascose synthesis [84,87]. Further, transfer of galactinol to verbascose via STS leads to ajugose [84,86,88]. In the above-mentioned RFO biosynthesis process, galactinol serves as a donor for galactosyl in each step, thus this route of RFO biosynthesis is referred to as galactinol-dependent pathway. However, another significant enzyme, galactan-galactosyltransferase (GGT), is involved in the galactinol independent route, which is less frequent than the first and has only been documented in two Lamiaceae species: *Coleus blumei* and *Ajuga reptans* [84,89–91]. The GGT catalyses the transfer of the galactosyl moiety from one RFO molecule to another, resulting in the formation of a higher RFO member. For instance, by the action of GGT, the transfer of a galactosyl moiety from one stachyose to another stachyose will result in the generation of verbascose and raffinose. In this manner, the GGT not only produces higher members of RFO but also modulates the concentration of cellular RFO [87,92,93].

**Figure 2:**
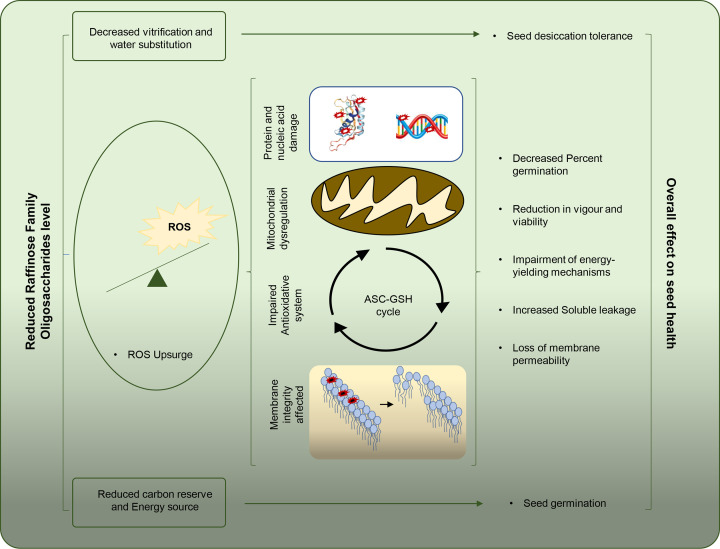
Role and the underlying mechanisms of RFOs in regulating the seed vigor and longevity or overall seed health

By the action of α-galactosidases RFOs are digested to sucrose and galactose. Sucrose breakdown into fructose and glucose by invertase or to UDP-glucose and fructose by sucrose synthase [84,93,94]. Galactose is first phosphorylated by the ATP-dependent galactokinase to form galactose-1-P (Gal-1P). Gal-1-P is further digested by two different pathways, one of which is the Lelior pathway and the other one is an alternative pathway in plants [95]. In the Lelior pathway, hexose-1-P uridylyltransferase transfers the UMP from UDP-glucose to galactose-1-phosphate resulting in UDP-galactose, with the release of glucose-1-phosphate [95,96]. However, in plants, galactose-1-phosphate is digested via an alternative pathway. Pyrophosphorylase converts galactose-1-phosphate and UTP to UDP-galactose and PPi. Subsequently, the NAD-dependent UDP-galactose-4-epimerase converts UDP-galactose to UDP-glucose [79,97]. The RFO biosynthetic pathway in plants involves some major enzymes including GolS, RAFS, STS, and VES whose manipulation in the crops leads to the acquiring of different stresses as well as other plant metabolic pathways [98–101] (Figure 2).

## Role of RFO in regulating plant seed vigor and longevity

Dry seeds should be disseminated from the mother plant to ensure plant generation continuation. When seeds grow, significant physiological, biochemical, and physical changes occur, resulting in the ability to survive in adverse environments [2]. Dehydration occurs at the end of the maturation phase in plant seeds, resulting in the accumulation of potentially protective compounds, notably soluble sugars like RFOs and sucrose, as well as LEA proteins [99,102,103]. The LEA proteins, in collaboration with the soluble sugars, contribute to the preservation of protein and membrane structural integrity in dry conditions by establishing a glassy state that prevents deteriorative processes [104,105]. As seeds expand and mature, they lose water, a process known as ‘desiccation’, in order to prepare for survival in harsh or unfavourable environments [2,106]. Non-reducing sugars such as sucrose and RFOs can build in seeds and prevent desiccation at that time to avoid harm, and numerous studies have revealed that RFOs have a role in desiccation tolerance. For instance, sucrose and RFOs, accumulate in the seeds of *Erythrina speciosa*, a Brazilian native tree, before significant changes in water content and are re-allocated from vacuole reserves to the cytosol late in seed maturation [107]. It has been postulated that they might help in maintaining the liquid crystalline condition of the cellular membranes in the dry state and are positively linked with desiccation tolerance as well as with seed longevity [108]. On the other hand, the Brazilwood seeds mainly show orthodox behavior and survive the desiccation during their maturation due to the accumulation of sugar alcohols like galactopinitol-A, galactopinitol-B, ciceritol, and lipids [109–111]. Broadly, two RFOs mechanisms have been suggested to control the desiccation process in seeds. The first mechanism is referred to as ‘vitrification’. This is the condition of a cell solution that has become very viscous owing to water loss. The cell solution has the characteristics of a plastic solid in this condition [42,112]. It is in control of cell stability, cellular collapse prevention, and hydrogen bonding preservation. LEA proteins, HSPs, and RFOs trigger the vitrification state [113]. The second is known as ‘water substitution’, in which RFOs’ hydroxyl groups can substitute water molecules within the cell and maintain the hydrophilic contacts required for the stability of native macromolecules and membrane structure during dehydration [112]. Similarly, these RFOs have a significant impact on seed germination, typically protect embryos from desiccation throughout seed maturation, and increase seed life under adverse conditions [6,22,114].

However, sugars are mostly postulated to operate as signalling molecules or as osmoprotectants; nevertheless, their role and accumulation, particularly RFOs, have been extensively researched in regulating and influencing seed vigor and longevity [101,115]. Like, RFOs have been demonstrated to aid sucrose in preserving membrane integrity by reducing lipid crystallization and aging processes [79,116,117]. Sucrose is the most abundant sugar in maize seeds, but its abundance did not correlate with enhanced storage; rather, raffinose as a mass proportion of total sugars showed a substantial and beneficial connection with seed vigor and longevity [6,90,118]. In soybean also it has been demonstrated that an increase in RFO to sucrose ratio as well as changes in RFO metabolism genes like GolS and RAFS has been positively associated with seed maturation, vigor, and longevity [119].

Additionally, changes in soluble sugar content, namely RFOs, have been associated with seed vigor and germinability in Arabidopsis and other species as well. Although the amount and type of RFOs that accumulates during seed maturity vary by species. For example, maize, Arabidopsis, and lettuce accumulate more raffinose than any other RFOs like stachyose and verbascose while castor bean accumulates more raffinose and stachyose but not verbascose. In contrast, galactinol and myo-inositol levels have been found too high in seeds across several species [90,120,121].

Legumes are the principal crops that acquire the most RFOs in their seeds [34]. Alpha-galactosides (α-Gal), sucrose-1,6-galactosyl derivatives, are one of the major complex sugars found in leguminous seeds. Furthermore, RFO accumulation and related α-GAL activity are linked to mature and germinating chickpea seeds [122]. When compared with the control, blocking RFO breakdown mediated by α-Gal with 1-deoxygalactonojirimycin (DGJ) reduced seed germination by approximately 25% in pea plants [123]. The accumulation of galactinol, and sucrose, occurs during the early stages of chickpea pod formation, while the raffinose, and stachyose, accumulate during the later stages of seed maturation, which suggested the accumulation of the higher-order RFOs pathway during seed maturation [100,114]. However, surprisingly, some research stated that low RFO genotypes of soybean [124] and chickpea [35,90] did not demonstrate delayed germination, suggesting that RFOs have no significant contribution to enhancing seed germination.

Similarly, galactinol synthesis by GolS from Myo-inositol and UDP-galactose is viewed as a crucial regulating step in RFO biosynthesis [31,125,126]. Carbon partitioning between sucrose and raffinose saccharides is regulated by GolS. The researchers revealed a QTL mapping in tomatoes that identified a co-locating QTL on chromosome 2 for galactinol content and seed lifespan. The findings revealed a positive association between galactinol content and seed lifespan in the three species studied, Arabidopsis, cabbage, and tomato, demonstrating that this link exists across Brassicaceae and beyond [101]. They used a reverse genetics strategy to explore the involvement of GolS enzymes in seed longevity, employing T-DNA knockout lines in genes encoding enzymes of this system such as *GolS1*, *GolS2*, *STC*, and *α*-*GAL*, as well as Arabidopsis overexpresses of the cucumber *GolS2* gene. The *gols2* mutant and the *gols1 gols2* double mutant exhibited the lowest seed galactinol concentration, resulting in the shortest seed life [12,35,101,121]. One more recent study has found the up-regulation of GolS1 in the degradome sequencing after long-term storage and controlled deterioration of *Nicotiana* and *Oryza sativa* seeds, which further correlates the RFO metabolism genes to seed longevity [127].

Collectively, RFOs are almost ubiquitous in the plant kingdom and may be found in a variety of vegetative organs and seeds. RFO accumulation in response to multiple biotic and abiotic stresses implies that RFOs have a function in stress adaptation in addition to acting as a signalling molecule and osmoprotectants during seed development and longevity [22]. In addition to its involvement in stress adaptation, *GolS* is involved in alkaloid tolerance and may play a role in cotton male fertility, fiber quality, and seed development [117]. Because GolS is a critical regulatory enzyme in RFO synthesis, various traditional and novel transgenic approaches have been explored to develop plants that are more resistant to stress as well as seed vigor and longevity [12,32,35]. The significance of RFOs in plant health is currently being debated, but it has several implications.

## Accumulation of RFOs in relation to acquisition of seed vigor and longevity

The RFOs content and the RFO-to-sucrose ratio have been used in several studies to predict seed quality and storability. The predominant soluble embryonic carbohydrate reserves in quiescent seeds are sucrose, with minor levels of oligosaccharides, raffinose, stachyose, and/or verbascose [6,36,117]. Changes in the soluble carbohydrate content, notably raffinose, may lead to both seed vigor and germinability losses. The analysis of sugar alterations during accelerated aging of maize seeds shows that the decrease in vigor is positively related to a significant decrease in monosaccharides and raffinose content [128]. Soluble carbohydrates are known to decrease with seed age, and this decrease may result in a lack of respiratory substrates for germination. Sucrose is one of the greatest vitrifying sugars and is extremely good at maintaining membrane integrity in dry environments [112]. However, raffinose is known to boost sucrose’s protective properties by reducing crystallization [112,129,130]. Pukacka et al. (2009) observed the correlation of RFOs content and its proportion with sucrose with the acquisition of seed desiccation tolerance and seed longevity in the beech. They have found that seeds stored at −10°C for the long term (in years) possess significantly high sucrose to RFOs ratio and α-Gal activity that has been negatively linked with seed germination capacity. While the accumulation of stachyose, an RFO member, helps in maintaining the seed viability for a longer period (Pukacka et al*.*, 2009). It was discovered that many enzymes, including α-Gal and invertase activity, were detectable at various levels in dried seeds [27,131]. In soybean also it has been demonstrated that an increase in RFO to sucrose ratio as well as changes in RFO metabolism genes has been positively associated with seed maturation, vigor, and longevity [119]. As GolS catalyses the first and most important step in the RFO biosynthesis pathway, which is a highly specialized metabolic mechanism in plants [12,90,101]. Salvi et al. (2016) observed that GolS activity and galactinol and raffinose concentration increase progressively as seed development progresses and become quite abundant in a pod and mature dry seeds but gradually decrease as seed germination progresses in chickpea seeds. A thorough examination of Arabidopsis *GolS* genes indicated that this enzyme also influenced seed longevity. In some studies, the concentration of galactinol in seeds has been positively associated with seed vigor and viability [20,101,132,133]. According to a recent study on *Oryza sativa*, PIMT is a well-known repairing enzyme that aids in the establishment of seed desiccation tolerance and seed longevity. In comparison with PIMT RNAi lines, PIMT overexpression lines with increased seed longevity had a higher accumulation of GolS transcript as well as galactinol content [20]. Being a protein repairing enzyme, PIMT repairs the transcriptional regulators and antioxidative enzymes, which maintain the cellular integrity during stress conditions and impart desiccation tolerance [20,68]. PIMT repairs the ABI-TF (ABA INSENSITIVE), which is a transcriptional regulator of the galactinol synthase gene [134]. Thus, the lines with an elevated level of PIMT possess adequate coordination of ABA and ABI-TF with higher galactinol levels to maintain improved seed vigor and longevity [20]. Within a certain genetic background, galactinol is undoubtedly a viable bio-marker of seed longevity, which may be useful to the seed industry in deciding which seed batches may be sold or preserved for longer periods [101]. These findings support the idea that sugar levels may fluctuate throughout seed storage and that estimating the sugar to RFO ratio and/or RFOs content can offer information regarding the viability and vigor of seeds [27,36,117,128] and GolS and RFO content increased seed vigor and longevity by decreasing age-induced excess ROS and lipid peroxidation.

## Challenges and future prospects to improve seed vigor and longevity through RFO manipulation

Climate change, expanding population, and consumer demand for high-quality food are all compelling researchers to explore the precise processes of seed viability and longevity in important agricultural plants [14]. Certainly, the development of advanced biotechnological technologies and omics tools has allowed us to gain a deeper understanding of the molecular mechanisms underlying seed vigor and viability [14,135]. Comparing cellular and metabolic changes throughout seed development, storage, and germination may provide useful information. Yet, the precise mechanism remains unknown and demands more research.

Several studies conducted over the last few decades have shed insight on the significance of sugar and its signalling in plant metabolism [27,28,80,90,92,113,117,136–138]. Sucrose, RFOs, and fructans have all been identified as carbon stores, membrane stabilizers, and anti-stress factors. RFOs hold a potential role in controlling seed vigor and longevity at varied levels [22]. According to one theory, RFOs may impede cytosolic crystallization in the dry state [112]. This cytoplasmic glass inhibits potentially harmful cellular responses, extending seed shelf life. However, more study is needed to identify the molecular mechanism of RFOs mediated protection of seeds against aging-induced ROS and how the RFOs:Suc ratio affects DNA damage and repair during germination [22].

Next, the significance of sugar transporters at the molecular level in modulating seed vigor and lifespan is largely unclear. Senescence is the final stage of a developmental program and is a carefully controlled event that involves the breakdown, removal, mobilization, or distribution of sugars or RFOs to other areas for storage. Such remobilization can be used for either growth and development or storage in the future by the tissues [139–143]. But how do sugar transporter’s function or act and the molecular mechanism underlying when seeds become mature or desiccated still need to be explored? Additionally, it would be very interesting to know how the signal has been perceived by the transporters to do this task.

The molecular players regulating seed vigor and longevity have been studied extensively in model plants such as *Arabidopsis thaliana* and *Medicago truncatula*, but not in cereal crops immensely. Although rice, tomato, and cotton were employed in different studies, they were all carried out in carefully controlled environments. As a result, the mechanism controlling seed longevity in economically important crop grains mainly cereals may be an interesting issue to explore in order to boost seed vigor in natural conditions [1,13,117]. Furthermore, the influence of overexpressed or knockout genes on nutritional value, genetically modified crop ethics, and public health risk may be the most significant problem that needs to be in mind in improving grain seed vigor and viability [1]. Because of the fast progress of biotechnological technologies, genome editing may be the best way for design-based crop enhancement by targeting critical agronomic factors like seed vigor and longevity [144].

Genome editing is an effective tool for hastening the production of cultivars with enhanced alleles, which have also been approved for distribution as a new variety in the public sector [145] The CRISPR/Cas9 system allows for precision genome editing in specific genes enabling the generation of genetically engineered crops, and it may be a helpful way for directly changing the genome to improve seed vigor and longevity [98]. This technique is appropriate for the targeted functional dissection of multiple genes, such as those involved in raffinose production and PLD activity, which are mainly responsible for causing seed destruction or deterioration during long-term storage. Recently, DNA binding with one finger transcription factor (DOF-TF) was found to be associated with RFO levels in Grapevine (Vitis vinifera). The overexpression lines of VaDof17d showed improved stress tolerance while the genome-edited lines displayed reduced RFO content and compromised growth under cold stress [146]. DOF-TF family is widely known for their role in abiotic stress responses in different crop plants [147–149], and thus can be used as a candidate molecular target for crop improvement. Similarly, knockout lines of a stress-responsive PP2C protein, i.e., OsPP65, generated through CRISPR/Cas9 showed improved tolerance against osmotic and salinity stress. OsPP65 modulate the abscisic acid and jasmonic acid signaling in rice, and the edited lines exhibited higher galactinol content under stress treatment as compared to their control counterpart [150]. Besides, in order to enhance the nutritional quality, the CRISPR/Cas9 approach has been used to reduce the RFO content of soybean. In the knockout soybean seeds, a substantial decline in stachyose content (35%) and increased raffinose content (42%) was observed. Interestingly, the edited soybean seeds did not show any significant difference in seed physiological traits such as morphology, germination, and development [151]. Moreover, CRISPR-mediated promoter editing and modified version of the CRISPR/Cas9 system like modified methylation at a specific CpG site are game-changing techniques that can completely interrupt targeted gene expression [144,152] and have potential as genome editing tools for site-specific epigenetic factor modification.

Recent advancements have offered vital insights into the actions of sugars and sugar polymers in seed vigor and the possibilities of boosting it by altering the composition of certain carbohydrates [9]. To understand the activity and expression profile under actual conditions, multilocation studies with the target crops are also required. Furthermore, QTLs and candidate genes discovered through mapping research might be used in breeding programs by utilizing genomic selection (GS), marker-assisted recurrent selection (MARS), and marker-assisted selection (MAS) [153] to increase the expression of the desired characteristics in economically important crops.

## Concluding remarks

Climate change reduced agricultural areas due to increased urbanization, and an ever-growing population that all threatened food security. Improving crop yield through traditional and next-generation breeding programs is a key strategy used by researchers to boost food production. Improved seed vigor and longevity is an essential characteristic that can ensure food and nutritional security for future generations. Seed longevity is an indicator of seed viability during long-term storage and is critical for germplasm conservation and agricultural enhancement initiatives [1]. However, traditional self-breeding frequently deteriorates seed quality as well as its viability, resulting in massive crop inferiority [77]. In addition, during storability, seed viability is reduced by different factors, including storage temperature, relative humidity, seed moisture content, biotic or microbe loads, etc. [9,28] Thus, a better knowledge of the different variables affecting seed life is required to increase its potential in this progressive world and prevent genetic drift during germplasm regeneration.

Carbon is often stored in plants as starch and translocated as sucrose [24]. Several plant species, however, can store and transport alternative carbohydrates like RFOs [117]. Sugars are stored in the seeds towards the end of the plant’s life cycle and give protection against desiccation damage during seed maturity as well as energy for the effective establishment of new seedlings during seed germination [106]. Clearly, the above-mentioned research shows that RFOs have an important role to play in seed maturation, desiccation, and germination. Furthermore, the activities of RFO biosynthesis enzymes like galactinol synthase, raffinose synthase, and stachyose synthase were shown to be positively regulated by seed longevity and vigor [12,83,92,132,154]. Even though several omics technologies have discovered seed longevity genetic variables along with several QTLs, their practical use in enhancing seed vigor and longevity through genetic engineering remains uncertain [101]. This creates a research gap and needs further attention to explore these possibilities for enhancing economically important crop seed attributes. Thus, in addition to improving storage facilities, boosting seed longevity by genetic manipulation of the identified genes has tremendous potential to ensure seed viability safety for a longer period. Manipulation of candidate genes, especially in cereals or economically important crops, has a significant impact on global food security. Reverse genetics combined with sophisticated gene-editing techniques, such as the CRISPR/Cas system [98,155,156], will give a potential approach to incorporate longevity qualities into major and minor cereal crop breeding efforts. Moreover, boosting seed longevity along with stress tolerance is crucial, as stress tolerance will not compensate for seed germination failure, which has a detrimental influence on grain yield and food security. In a nutshell, the advancement in molecular tools, next-generation omics techniques, and modern breeding strategies offer enormous potential for improving seed vigor and longevity in economically important crops. Improved cultivars with higher quality and lifespan features can assure food and nutritional security for the world’s expanding population and climate changes.

## Data Availability

Data sharing does not apply to this article as no new data were created or analysed in this study.

## Abbreviations

ABI: abscisic acid insensitive
DGJ: 1-deoxygalactonojirimycin
DT: desiccation tolerance
GGT: galactan-galactosyltransferase
GolS: galactinol synthase
GS: genomic selection
MARS: marker-assisted recurrent selection
MAS: marker-assisted selection
PIMT: protein l-isoaspartyl methyltransferase
RAFS: raffinose synthase
RFO: raffinose family oligosaccharide
STS: stachyose synthase
VES: verbascose synthase
